# Retraction: Long non-coding RNA HAL suppresses the migration and invasion of serous ovarian cancer by inhibiting EMT signaling pathway

**DOI:** 10.1042/BSR-20194496_RET

**Published:** 2021-09-13

**Keywords:** LncRNA HAL, migration, proliferation, serous ovarian cancer

This article is being retracted from *Bioscience Reports* at the request of the Editor-in-Chief and the Editorial Board following receipt of a notification from a reader, alerting the Editorial Board to the tumor images inf Figure 6B, which have been published in multiple publications by different authors.

The authors have been contacted in regards to the Retraction, and have not responded to the Journal's queries and the concerns raised. Given the extent of the issues raised, the Editorial Board stand by the decision to retract the article.

